# *Carpinus tibetana* (Betulaceae), a new species from southeast Tibet, China

**DOI:** 10.3897/phytokeys.98.23639

**Published:** 2018-05-02

**Authors:** Zhiqiang Lu, Ying Li, Xiaoyue Yang, Jianquan Liu

**Affiliations:** 1 State Key Laboratory of Grassland Agro-Ecosystem, College of Life Science, Lanzhou University, Lanzhou 730000, China; 2 Key Laboratory for Bio-resources and Eco-environment of Ministry of Education, College of Life Science, Sichuan University, Chengdu 610064, China; 3 Key Laboratory of Tropical Forest Ecology, Xishuangbanna Tropical Botanical Garden, Chinese Academy of Sciences, Yunnan 666303, China

**Keywords:** *Carpinus tibetana*, new species, Tibet

## Abstract

A new species *Carpinus tibetana* Z. Qiang Lu & J. Quan Liu from southeast Tibet is described and illustrated. The specimens of this new species were previously identified and placed under *C. monbeigiana* Hand.-Mazz. or *C. mollicoma* Hu. However, the specimens from southeast Tibet differ from those of *C. monbeigiana* from other regions with more lateral veins (19–24 vs 14–18) on each side of the midvein and dense pubescence on the abaxial leaf surface, while from those of *C. mollicoma* from other regions differ by nutlet with dense resinous glands and glabrous or sparsely villous at apex. Principal Component Analyses based on morphometric characters recognise the Tibetan populations as a separate group. Nuclear ribosomal ITS sequence variations show stable and distinct genetic divergences between the Tibetan populations and *C. monbeigiana* or *C. mollicoma* by two or three fixed nucleotide mutations. Phylogenetic analysis also identified three respective genetic clusters and the *C. mollicoma* cluster diverged early. In addition, the Tibetan populations show a disjunct geographic isolation from the other two species. Therefore, *C. tibetana*, based on the Tibetan populations, is here erected as a new species, distinctly different from *C. monbeigiana* and *C. mollicoma*.

## Introduction

**Figure 1.**
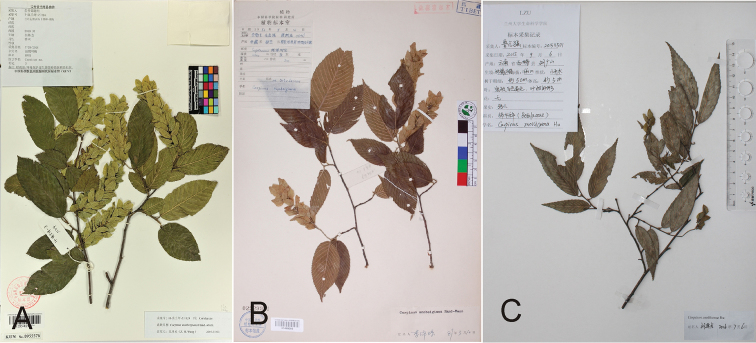
The gross morphology of two specimens had been identified as *Carpinus monbeigiana* and one as *C. mollicoma*. **A** *C. monbeigiana* from Yunnan (*H. Peng et al. H-Lanping-Z1124*, KUN) **B** *C. monbeigiana* from Tibet (*B.S. Li et al. 6467*, PE) **C** *C. mollicoma* from Yunnan (*Z.Q. Lu 201511501*, LZU). The number of the lateral leaf veins is totally different between two specimens from Yunnan and Tibet (**A** and **B**).

The birch family (Betulaceae) comprises six genera and approximately 167 species (Christenhusz and Byng 2016). In this family, the hornbeams in the genus *Carpinus* (Linnaeus, 1753) are small to medium-size trees (Li and Skvortsov 1999; Holstein and Weigend 2017). In *Flora of China*, 33 hornbeam species are described and 28 of which are endemic (Li and Skvortsov 1999). The endemic species *C. monbeigiana* Hand.-Mazz. is mainly distributed in southeast (SE) Tibet and northwest (NW) Yunnan. This species is recognised due to the leaves doubly or simply setiform serrate along the margin, nutlets with dense resinous glands, peduncles and rachises with densely yellow hirsute and densely hispidulous bracts with an inflexed auricle at the base of the inner margin. However, a small number of specimens from SE Tibet were also identified as *C. mollicoma* Hu because of the numerous lateral veins and dense pubescence on the abaxial leaf surface (Li and Skvortsov 1999). Another species, *C. viminea* Wall. ex Lindl. is also distributed to SE Tibet and NW Yunnan (Wu 1991, Li and Skvortsov 1999). However, *C. viminea* is distinctly different from both *C. monbeigiana* and *C. mollicoma* with the long leaf petiole and a lobe at the base of the inner margin of bract. After examining all specimens of *C. monbeigiana* and *C. mollicoma* preserved in the Chinese Virtual Herbarium (http://www.cvh.org.cn) and Lanzhou University (LZU) in 2015, we found that the specimens from Tibet under *C. monbeigiana* or *C. mollicoma* might stand as a new species because they are clearly different from specimens of the two species collected from Yunnan (Figure 1). In order to further test this hypothesis, we conducted field surveys and an examination of morphological variation and genetic divergence. All lines of evidence support the establishment of a new species to accommodate the Tibetan populations as distinct from both *C. monbeigiana* and *C. mollicoma*.

## Material and methods

### Field surveys

After examining *Carpinus* specimens preserved in KUN and PE (Table 1), we found that the nutlet sizes of *C. monbeigiana* become stable after July. This was further confirmed by the measurements of the nutlet sizes of *C. monbeigiana* collected between July and September in 2015 from the same locality (Xishan, Kunming, Yunnan Province). Hence, collections from before July were excluded in our measurements of the morphological variation of specimens. We conducted the field surveys in Tibet and Yunnan from July to September in 2015 and 2016 in order to collect enough samples from different individual trees for morphological analyses and later genetic analyses. For the latter purpose, fresh leaves of each tree were immediately dried by silica gel in a plastic bag. All sampled populations of *C. monbeigiana* and *C. mollicoma* in the field are listed in Table 2. Voucher specimens were deposited in Lanzhou University Herbarium (**LZU**).

**Table 1.** Specimens used for Principal Component Analyses (PCA) of morphological variations.

**Species**	**Collector**	**Collection number**	**Collection site**	**Herbarium**	**No. of specimen**
*C. tibetana*	*B.S. Li*	*06467/6467*	Yigong, Linzhi, Xizang	PE	2
*C. tibetana*	*W.L. Chen*	*10780*	Motuo, Xizang	PE	1
*C. tibetana*	*Anonymous*	*15079*	Ani to Hanmi, Motuo, Xizang	PE	1
*C. tibetana*	*Wu*	*5649*	Yigong, Bomi, Xizang	KUN	1
*C. tibetana*	*Anonymous*	*2505*	Tongmai, Bomi, Xizang	PE	1
*C. tibetana*	*H. Sun et al.*	*SunH-07ZX-2725*	Yigong, Bomi, Xizang	KUN	1
*C. tibetana*	*H. Sun et al.*	*6008*	Dexing, Motuo, Xizang	PE	1
*C. tibetana*	*Z.Q. Lu*	*2016QTP001- 2016QTP011*	Tongmai, Bomi, Xizang	LZU	11
*C. mollicoma*	*Z.Q. Lu*	*201511501*-*201511517*	Xisha, Xichou, Yunnan	LZU	17
*C. monbeigiana*	*G.M. Feng*	*23645*	Huanfuping, Deqin, Yunnan	KUN	1
*C. monbeigiana*	*G.M. Feng*	*21595*	Jiazi, Lijiang, Yunnan	PE	1
*C. monbeigiana*	*G.M. Feng*	*50081/10121*	Xishan, Kunming, Yunnan	KUN	2
*C. monbeigiana*	*X.H. Yang*	*101202*	Xishan, Kunming,Yunnan	KUN	1
*C. monbeigiana*	*Z.Q. Lu*	*2015KM001-2015KM005*	Xishan, Kunming,Yunnan	LZU	5
*C. monbeigiana*	*W.Z. Li*	*147/149*	Xishan, Kunming,Yunnan	CSFI	2
*C. monbeigiana*	*Anonymous*	*30081*	Xishan, Kunming, Yunnan	KUN	1
*C. monbeigiana*	*Q.W. Wang*	*66847/67245*	Dela, Gongshan,Yunnan	PE	2
*C. monbeigiana*	*Anonymous*	*7340/7935/7940/ 7950/7954/8024*	Bingzhongluo, Gongshan,Yunnan	PE	6
*C. monbeigiana*	*Anonymous*	*22012*	Pengdang, Gongshan,Yunnan	KUN	1
*C. monbeigiana*	*T.T. Yu*	*19184*	Gongshan, Yunnan	PE	1
*C. monbeigiana*	*T.T. Yu*	*19103*	Mekong-Salwin divide, Gongshan, Yunnan	PE	1
*C. monbeigiana*	*Anonymous*	*22904*	Mekong-Salwin divide, Gongshan, Yunnan	KUN	1
*C. monbeigiana*	*S.D. Liu et al.*	*03-103*	Wumulong, Yongde, Yunnan	KUN	1
*C. monbeigiana*	*H. Peng et al.*	*H-LP-Z1124*	Tongdian, Lanping, Kunming	KUN	1
*C. monbeigiana*	*Z.Q. Lu*	*2016WXYZ001- 2016WXYZ019*	Yezhi, Weixi, Yunnan	LZU	19
*C. monbeigiana*	*Z.Q. Lu*	*2016WXKP001-2016WXKP005*	Kangpu, Weixi, Yunnan	LZU	5
*C. monbeigiana*	*P.Y. Mao*	*00356/00370/00836*	Kangpu, Weixi, Yunnan	PE	3

**Table 2.** Locations of the sampled populations from which individuals were used for genetic analyses of the nuclear ribosomal ITS sequence variations.

**Species (individual number)**	**Location**	**Latitude (N)**	**Longitude (E)**	**Altitude (m)**
*C. tibetana* (6)	Tongmai, Bomi, Tibet	30°06'N, 95°05'E		2060
*C. tibetana* (2)	Tongmai, Bomi, Tibet	30°01'N, 95°03'E		2080
*C. monbeigiana* (5)	Xishan, Kunming, Yunnan	24°58'N, 102°38'E		2355
*C. monbeigiana* (8)	Yezhi, Weixi, Yunnan	27°48'N, 99°02'E		1790
*C. monbeigiana* (2)	Kangpu, Weixi, Yunnan	27°38'N, 99°01'E		1660
*C. monbeigiana* (1)	Weideng, Weixi, Yunnan	27°06N, 99°07'E		1685
*C. mollicoma* (9)	Xisha, Xichou, Yunnan	23°26'N, 104°40'E		1660

### Morphological analysis

A total of 90 specimens (19 from southeast Tibet, 17 for *C. mollicoma* and 54 for *C. monbeigiana*) from individual trees were used for morphological comparisons. We examined morphological variations within and between the Tibetan populations and *C. monbeigiana* and *C. mollicoma* from other regions (Table 1) and measured 22 characters for morphological Principal Component Analyses (PCA) (Table 3).

**Table 3.** Morphological characters of *C. tibetana*, *C. monbeigiana* and *C. mollicoma* at the population level.

**Characters**	***C. mollicoma***	***C. tibetana***	***C. monbeigiana***
**LEAF**			
Shape and size	Leaf blade oblong-lanceolate, or elliptic-lanceolate, rarely ovate-lanceolate, 4.5–8 cm × 1.5–3 cm; apex acute, acuminate or caudate-acuminate	Leaf blade ovate-elliptic or elliptic, 6–9 cm × 3–4 cm; apex attenuate-acuminate or caudate-acuminate	Leaf blade oblong-lanceolate, ovate-lanceolate, or elliptic-lanceolate, 6–13 cm × 3–4.5 cm; apex acute, acuminate, rarely caudate-acuminate
Length of petiole	3–8 mm	5–8 mm	6–12 mm
Number of lateral veins on each side of midvein	15–21	19–24	14–18
Average distance between lateral veins located in the middle of leaf	4–5 mm	4–5 mm	5–8 mm
Abaxially densely pubescent or glabrescent	Densely pubescent	Densely pubescent	Usually glabrescent
**INFRUCTESCENCE**			
Size of infructescence	2.5–4.5 cm × 1–1.5 cm; peduncle 1–1.5 cm	4–7 cm × 1.5–2.5 cm; peduncle 1–2.5 cm	4–13 cm × 1.5–3 cm; peduncle 1–3 cm
**BRACT**			
Size of bract	0.9–1.9 cm × 0.4–0.6 cm	1.5–1.9 cm × 0.6–0.9 cm	1.2–2.3 cm × 0.5–1.2 cm
**NUTLET**			
The number of ribs	6–9	7–11	6–10
Densely villous or glabrous	Densely villous	glabrous or sparsely villous at apex	glabrous or sparsely villous at apex
Densely resinous glandular or not	Not	Densely resinous glandular	Densely resinous glandular
Shape and size of nutlet	Broadly ovoid orovoid-ellipsoid, 3.1–3.7 mm × 2–2.6 mm	Ovoid-ellipsoid, 3.0–3.9 mm × 2.2–2.8 mm	Broadly ovoid, 3.2–4.6 mm × 2.9–4.1 mm

### Genetic analysis

For genetic analyses of the nuclear ITS region, 33 individuals from 7 populations of three groups were used. Amongst them, 8 individuals from two populations were collected from southeast Tibet while 9 individuals for *C. mollicoma* and 16 individuals for *C. monbeigiana*. *Carpinus viminea* was also included because this species also occurs in SE Tibet and NW Yunnan (Wu 1991, Li and Skvortsov 1999). Total DNA was extracted from 15–25 mg silica gel dried leaves using the modified CTAB method (Doyle and Doyle 1990). Nuclear ribosomal ITS sequence was used to confirm the species status of the Tibetan populations because the sequence variation of this fragment is stable within and between species with high species discrimination power (Lu et al. 2016). PCR amplifying and sequencing of the ITS fragment followed Lu et al. (2016). All newly available ITS sequences were uploaded to GenBank under the accession numbers KY436145–KY436155 and KY683787–KY683789. We used RAxML-8.1.17 (Stamatakis 2014) to conduct the Maximum likelihood (ML) analyses under the GTR + G model. Bootstrap replicates (1000) were set to calculate the support values.

## Results

**Figure 2.**
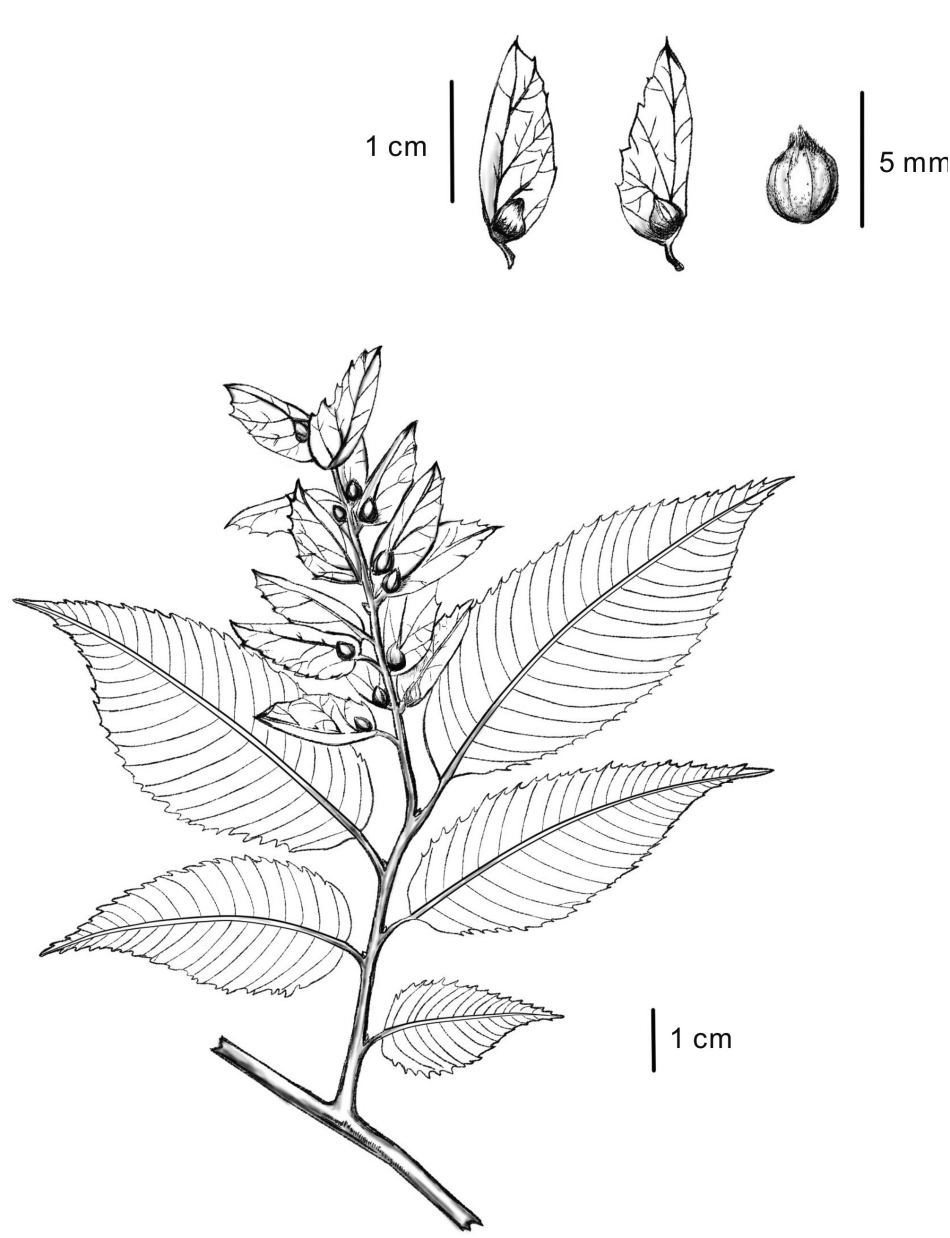
*Carpinus tibetana* Z. Qiang Lu & J. Quan Liu was drawn from *Z.Q. Lu 2016QTP001* (LZU).

**Figure 3.**
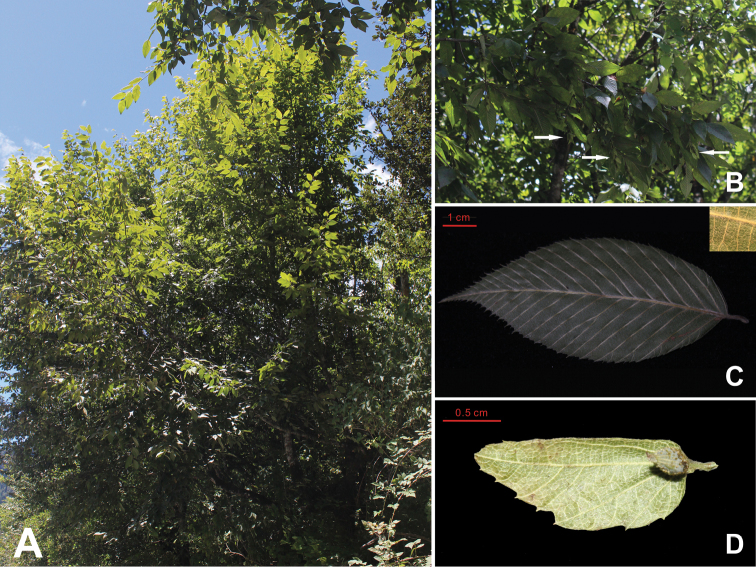
*Carpinus tibetana* Z. Qiang Lu & J. Quan Liu. **A** The whole plant **B** Branches with infructescences, the small white arrows pointing to the infructescences **C** Leaf **D** Bract and fruit.

**Figure 4.**
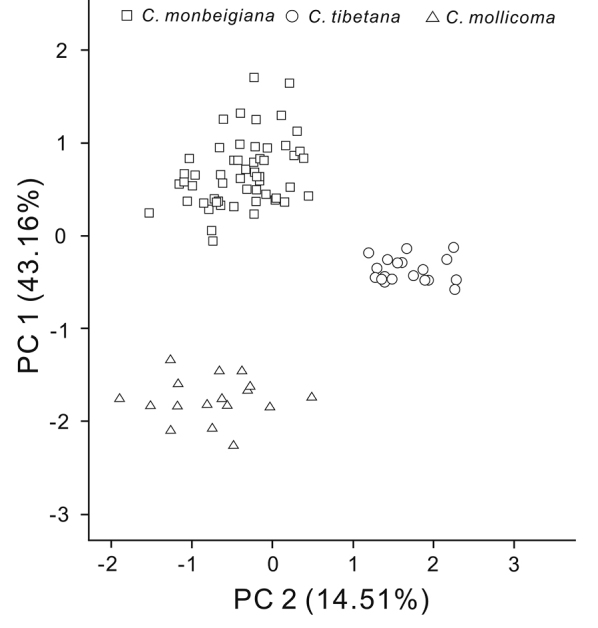
Morphological clustering based on Principal Component Analyses separated three different groups responding to the respective species.

Morphologically, the Tibetan populations (Table 1; Figures 2–3) differ distinctly from those of *C. monbeigiana* from Yunnan with more lateral veins (19–24 vs 14–18) on each side of the midvein and more densely pubescent on the abaxial leaf surface and the difference was also found in the narrower distance between lateral veins (4–5 mm vs 5–8 mm) and smaller nutlet (Table 3). Meanwhile, plants of the Tibetan populations also differ from *C. mollicoma* by the nutlet having dense resinous glands and being glabrous or sparsely villous at apex. The difference was also found in the size of infructescence (2.5–4.5 cm × 1–1.5 cm vs 4–7 cm × 1.5–2.5) and bract (0.9–1.9 cm × 0.4–0.6 cm vs 1.5–1.9 cm × 0.6–0.9 cm). A Principal Component Analyses (PCA) distinguished samples from the two species and the Tibetan populations into three different groups (Table 4; Figure 4). The first principal component axis (PC1; accounting for 43.16% of the variation) significantly separated *C. mollicoma* from *C. monbeigiana* and Tibetan populations, where there was a slight overlap between them. However, the second principal component axis (PC2; 14.51%) significantly separated the Tibetan populations from the other two species.

**Table 4.** Morphological characters measured for Principal Component Analysis (PCA).

**Character number**	**State**	**Unit**	**Coding (if qualitative)**	**PC1 (43.16%)**	**PC2 (15.51%)**
	**LEAF**		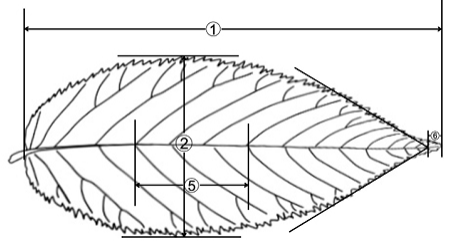		
1	Length	cm		0.672	0.316
2	Width	cm		0.783	0.432
3	Length to width ratio	Ratio		-0.584	-0.366
4	Length of petiole	cm		0.224	-0.246
5	Distance between 5-8 lateral veins located in the middle of leaf	cm		0.853	-0.196
6	Length of apex	mm		-0.357	0.633
7	Average petiole diameter in the middle	mm		0.592	-0.094
8	Character1/ Character5	Ratio		0.544	0.703
9	Number of lateral veins on each side of midvein	Count		-0.488	0.754
10	Abaxial leaf pubescence	Qualitative	2 = Dense; 1 = Glabrescent	-0.694	0.308
	**INFRUCTESCENCE**				
11	Length of peduncle	cm		0.690	0.250
12	Length of infructescence	cm		0.754	-0.430
13	Width of infructescence.	cm		0.736	0.174
	**BRACT**				
14	Length	cm		0.697	0.283
15	Width	cm		0.663	0.377
16	Length to width ratio	Ratio		-0.410	-0.195
	**NUTLET**				
17	Densely villous or not	Qualitative	2 = Dense; 1 = None or sparsely villous at apex	0.856	0.360
18	Densely resinous glandular or not	Qualitative	2 = Dense; 1 = None	-0.856	-0.360
19	Number of ribs	Count		0.189	0.434
20	Length of nutlet	cm		0.586	-0.511
21	Width of nutlet	cm		0.861	-0.344
22	Length to width ratio	Ratio		-0.884	-0.160

**Figure 5.**
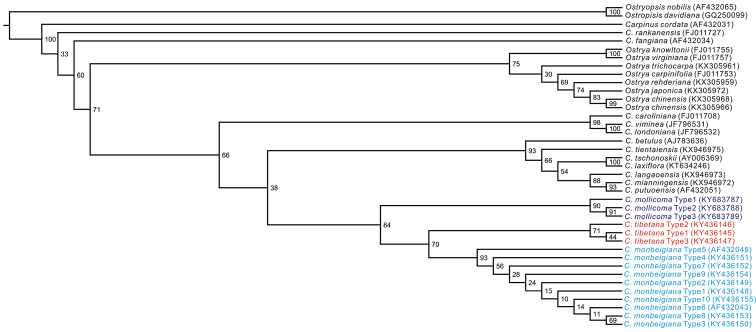
The ML tree based on nuclear ribosomal ITS sequence data from related species. GenBank accession numbers are shown after each species name.

Genetically, the aligned 33 ITS sequences were 611 base pairs in length. In addition, three ITS sequences from *C. monbeigiana* were also downloaded from NCBI (AF432043, AF432044 and AF432048). In total, 16 types were identified from these sequences and the individual number of shared types is presented in Table 5. Phylogenetic analysis of these sequences suggested that the sampled individuals of *C. monbeigiana*, *C. mollicoma* and the Tibetan populations separated into three genetic clades with *C. mollicoma* diverging first and *C. monbeigiana* and plants from the Tibetan populations forming a sister relationship (Figure 5). The sequence variations of the Tibetan individuals showed a combination of the mutations found for *C. mollicoma* or *C. monbeigiana* (Table 2).

**Table 5.** Nuclear ribosomal ITS sequence variations between three closely related species. The fixed nucleotide mutations were presented in bold type. Three ITS sequences (Type 5 and Type 6) of *Carpinus monbeigiana* (from Yunnan) were downloaded from NCBI (AF432043, AF432044 and AF432048).

	19 variable positions																		
Types of ITS sequences	1	1	1	1	1	1	1	3	3	3	4	4	4	4	5	5	5	5	5
(Individual number of the shared types)	9	4	5	8	8	8	9	0	9	9	2	2	4	5	4	5	5	5	8
		6	1	0	4	9	9	3	1	6	7	8	0	5	5	4	5	6	0
*C. tibetana* Type1 (5)	**A**	A	G	G	A	C	T	G	A	T	G	T	C	A	G	C	T	G	**G**
*C. tibetana* Type2 (2)	**A**	A	G	G	A	C	T	G	A	T	G	T	C	A	G	C	W	G	**G**
*C. tibetana* Type3 (1)	**A**	A	G	G	A	C	T	G	A	Y	G	T	C	A	G	C	T	G	**G**
*C. monbeigiana* Type1 (4)	G	A	G	R	A	Y	Y	G	R	T	S	K	Y	A	S	Y	T	G	A
*C. monbeigiana* Type2 (4)	G	A	G	R	A	Y	Y	G	R	T	S	K	Y	A	S	C	T	G	A
*C. monbeigiana* Type3 (2)	G	A	G	G	A	Y	C	R	A	T	G	T	Y	A	G	C	T	G	A
*C. monbeigiana* Type4 (2)	G	A	G	G	A	Y	Y	G	A	T	G	T	T	A	G	C	T	G	A
*C. monbeigiana* Type5 (2)	G	A	G	G	A	C	T	G	A	T	G	T	T	A	G	C	T	G	A
*C. monbeigiana* Type6 (1)	G	A	G	G	A	T	T	G	A	T	G	T	T	A	G	C	T	G	A
*C. monbeigiana* Type7 (1)	G	A	G	G	A	Y	Y	G	R	T	G	T	T	A	G	C	T	G	A
*C. monbeigiana* Type8 (1)	G	A	G	G	A	Y	C	R	A	T	G	T	Y	A	G	C	T	G	A
*C. monbeigiana* Type9 (1)	G	A	G	G	A	Y	Y	G	R	T	G	T	Y	A	S	C	T	G	A
*C. monbeigiana* Type10 (1)	G	A	G	R	A	Y	Y	R	R	T	S	K	Y	A	S	Y	T	G	A
*C. mollicoma* Type1 (5)	**A**	A	**C**	A	**G**	C	T	G	A	T	G	T	C	**G**	G	C	T	G	**G**
*C. mollicoma* Type2 (2)	**A**	R	**C**	A	**G**	C	T	G	A	T	G	T	C	**G**	G	C	T	G	**G**
*C. mollicoma* Type3 (2)	**A**	G	**C**	A	**G**	C	T	G	A	T	G	T	C	**G**	G	C	T	R	**G**

**Figure 6.**
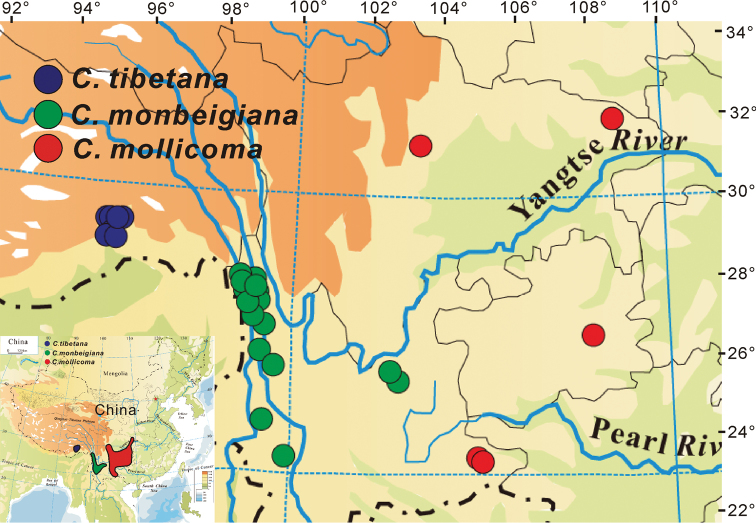
The distributions and locations of *C. monbeigiana*, *C. mollicoma* and *C. tibetana* based on the field investigation and Chinese Virtual Herbarium (http://www.cvh.org.cn/).

Geographically, all specimen records in the present study and those from Chinese Virtual Herbarium (http://www.cvh.org.cn/) suggested that the Tibetan populations are disjunct in geographical distributions from both *C. monbeigiana* and *C. mollicoma* (Figure 6).

## Discussion

Different species concepts emphasise the different criteria used to define and publish a new species (Wheeler and Meier 2000). An integrative practice using multiple criteria to circumscribe species boundaries will produce relatively objective and operational taxonomy (Su et al. 2015, Hu et al. 2015, Liu 2016; Lu et al. 2017). In this study, we demonstrated that the Tibetan populations previously placed under *C. monbeigiana* or *C. mollicoma* should be erected as a distinct new species based on the following lines of evidence. Firstly, these populations were obviously distinct from those of *C. monbeigiana* by the more lateral veins and dense pubescence on the abaxial leaf and from *C. mollicoma* by the nutlet with dense resinous glands and glabrous or sparsely villous at apex. All statistical analyses of the morphological traits clustered them into three separated groups. These populations seem to be characterised by a morphological combination of the other two species. Secondly, genetic divergences amongst these three groups are distinct; all of the sampled individuals from the Tibetan populations have a combination of unique genetic mutations that are found in the other two species but in a combination that is distinct from them. Phylogenetic analysis of nuclear ribosomal ITS sequence variations suggested that all sampled individuals from the Tibetan populations comprised a genetic cluster which seems to be more closely related to *C. monbeigiana* than to *C. mollicoma*. Finally, the Tibetan populations occupy a distinct distribution disjunct from others of *C. monbeigiana* and *C. mollicoma*. All lines of evidence suggest that the divergence amongst these populations is consistent with warranting three distinct species. Given this, we here recognise the Tibetan populations as a new species. In addition, this new species probably originated through the geographic isolation from hybrid (homoploid or allopolyploid) speciation between *C. monbeigiana* and *C. mollicoma* because of its morphological and genetic combination of the other two species. However, this hypothesis needs further tests from multiple lines of evidence, including both chromosomal and population genetic observations.

## Taxonomic treatment

### Carpinus tibetana

Taxon classificationPlantaeFagalesBetulaceae

Z. Qiang Lu & J. Quan Liu sp. nov.

urn:lsid:ipni.org:names:60476297-2

Figures 2 , 3

#### Diagnosis.

*Carpinus tibetana* differs from *C. monbeigiana* by 19–24 lateral veins on each side of the midvein and dense pubescence on the abaxial leaf and from *C. mollicoma* by the nutlet with dense resinous glands and glabrous or sparsely villous at apex.

#### Type.

CHINA. Tibet: Bomi County, Yigong, Tongmai, 95°04'31"E, 30°06'05"N, 2060 m, forest edge, 28 Aug 2016, *Z.Q. Lu 2016QTP001* (holotype, LZU; isotypes, LZU, PE, KUN).

#### Description.

Trees to 10 m tall, deciduous; bark grey, smooth. Branchlets dark grey, densely yellow or white pubescent when young, glabrescent the next year. Stipules deciduous. Petiole 5–8 mm, densely white or yellow puescent; leaves alternate, leaf blade ovate-elliptic or elliptic, usually 5–8 cm × 2–3 cm, abaxially sericeous-villous along veins, pubescent elsewhere, base rounded or rounded-cuneate, margin irregularly and doubly setiform mucronate serrate, apex attenuate-acuminate or caudate-acuminate; lateral veins (17) 19–23 on each side of midvein. Male inflorescence pendulous, spicate-cymose, cylindric, enclosed by buds during winter, with many overlapping bracts, 1.5–5.0 cm × 5.0–8.0 mm when mature; flowers without bracteoles, inserted at base of bracts. Female inflorescence terminal or axillary on dwarf shoots, racemose; flowers paired; bracts leaflike, complanate, overlapping. Mature infructescence 5–10 cm × 2.0–3.5 cm; peduncle ca. 1.2 cm, densely yellow hirsute; bracts of female flowers loosely overlapping, 1.5–1.9 cm × 6–8 mm, abaxially densely yellow hirsute along reticulate veins, outer margin coarsely dentate, without basal lobe, inner margin entire, with inflexed basal auricle, apex acuminate or caudate-acuminate; veins 5–6. Nutlet ovoid-ellipsoid, 3.2–3.6 mm × 2.2–2.5 mm, glabrous or sparsely villous at apex, densely brown or orange resinous glandular, prominently 8- or 9-ribbed. Fl. Apr–May, fr. Jul–Sep.

#### Etymology.

Due to its narrow distribution in Tibet, we give the specific epithet (*Carpinus tibetana*) referring to the name of the Xizang Autonomous Region (Tibet) of China where it is distributed.

#### Phenology.

Flowering from April to May and fruiting from May to September.

#### Habitat and distribution.

Up to now, according to our field surveys and sampling records in Chinese Virtual Herbarium (CVH), *Carpinus tibetana* has only been collected in Bomi and Motuo Counties (Figure 4). The new species usually grows at the forest edge and miscellaneous wood forest at elevations from 1550–2300 m a.s.l. This species probably extends its distribution to other Himalayan and adjacent regions in India, Nepal and Bhutan. Therefore, the *Carpinus* specimens collected from these regions need to be examined and confirmed and further field investigations to these regions should be conducted.

#### Additional specimens examined.

**CHINA. Tibet**: Linzhi City, Yigong River, forest edge, 2300 m, 8 Aug 1983, *B.S. Li et al. 06467 & 6467* (PE); Bomi County, near to Yigong Town, secondary forest, 2100 m, 8 Sep 1976, *Wu 5649* (PE); Bomi County, Tongmai, mixed forest, 2080 m, 24 Jun 1976, *Anonymous 2505* (PE); Bomi County, Tongmai to Lulang along the G318 National Road, forest edge, 95°00'48'' E, 30°02'35"N, 2060 m, 26 Sep 2009, *H. Sun et al. SunH-07ZX-2725* (KUN); Motuo County, Dexing, 26 Apr 1993, *H. Sun et al. 6008* (PE); Motuo County, from Ani to Hanmi, forest edge, 1550 m, 19 Sep 1980, *Anonymous 15079* (PE); Motuo County, forest edge, 1500 m, 29 Jun 1980, *W.L. Chen 10780* (PE); Bomi County, Yigong, Tongmai, 95°04'31"E, 30°06'05"N, 2060 m, forest edge, 28 Aug 2016, *Z.Q. Lu 2016QTP002*–*Z.Q. Lu 2016QTP011* (LZU).

##### Key for identification of these four related species in Yunnan and Tibet, China

**Table:** 

1	Bracts with lobes at bases of inner and outer margins; petioles slender, (1.0–)1.5–3.0 cm	***C. viminea***
–	Bracts with an inflexed auricle at base of inner margin; petioles robust, 0.3–1.2 cm	**2**
2	Infructescences 4–13 cm × 1.5–3 cm; bracts 1.2–2.3 cm × 0.5–1.2 cm; nutlets ovoid-ellipsoid or broadly ovoid, with dense resinous glands, glabrous or sparsely villous at apex	**3**
–	Infructescence 2.5–4.5 cm × 1–1.5 cm; bracts 0.9–1.9 cm × 0.4–0.6 cm; nutlets broadly ovoid or ovoid-ellipsoid, without resinous glands, densely villous	***C. mollicoma***
3	Leaf blade oblong-lanceolate, ovate-lanceolate, or elliptic-lanceolate, abaxial leaf surface glabrescent, with 14–18 lateral veins on each side of midvein, average distance between lateral veins 5–8 mm; nutlets broadly ovoid, 3.2–4.6 mm × 2.9–4.1 mm	***C. monbeigiana***
–	Leaf blade ovate-elliptic or elliptic, abaxial leaf surface densely pubescent, with 19–24 lateral veins on each side of midvein, average distance between lateral veins 4–5 mm; nutlets ovoid-ellipsoid, 3.0–3.9 mm × 2.2–2.8 mm	***C. tibetana***

## Supplementary Material

XML Treatment for Carpinus tibetana

## References

[B1] Christenhusz MJM Byng JW (2016) The number of known plants species in the world and its annual increase. Phytotaxa 261(3): 201–217. https://doi.org/10.11646/phytotaxa.261.3.1

[B2] Doyle JJ Doyle JL (1990) Isolation of DNA from small amounts of plant tissues. BRL Focus 12: 13–15.

[B3] Holstein N Weigend M (2017) No taxon left behind?–a critical taxonomic checklist of *Carpinus* and *Ostrya* (Coryloideae, Betulaceae). European Journal of Taxonomy 375: 1–52.

[B4] Hu H Al-Shehbaz IA Sun YS Hao GQ Wang Q Liu JQ (2015) Species delimitation in *Orychophragmus* (Brassicaceae) based on chloroplast and nuclear DNA barcodes. Taxon 64(4): 714–726. https://doi.org/10.12705/644.4

[B5] Li PC Skvortsov AK (1999) Betulaceae. In: Wu C Y, Raven PH (Eds) Flora of China, vol 4. Science Press, Beijing. 286–313.

[B6] Liu JQ (2016) ‘The integrative species concept' and ‘species on the speciation way'. Shengwu Duoyangxing 24(9): 1004–1008. https://doi.org/10.17520/biods.2016222

[B7] Lu ZQ Liu SY Yang XY Liang QL Yang YZ Zhang D Milne R Liu JQ (2017) *Carpinus langaoensis* (Betulaceae), a new hornbeam species from the Daba Mountains in Shaanxi, China. Phytotaxa 295(2): 185–193. https://doi.org/10.11646/phytotaxa.295.2.6

[B8] Lu ZQ Zhang D Liu SY Yang XY Liu X Liu JQ (2016) Species delimitation of Chinese hop-hornbeams based on molecular and morphological evidence. Ecology and Evolution 6(14): 4731–4740. https://doi.org/10.1002/ece3.2251 27547308 10.1002/ece3.2251 PMC4979702

[B9] Stamatakis A (2014) RAxML version 8: A tool for phylogenetic analysis and post-analysis of large phylogenies. Bioinformatics (Oxford, England) 30(9): 1312–1313. https://doi.org/10.1093/bioinformatics/btu033 10.1093/bioinformatics/btu033 PMC3998144 24451623

[B10] Su X Wu GL Li LL Liu JQ (2015) Species delimitation in plants using the Qinghai–Tibet Plateau endemic *Orinus* (Poaceae: Tridentinae) as an example. Annals of Botany 116(1): 35–48. https://doi.org/10.1093/aob/mcv062 25987712 10.1093/aob/mcv062 PMC4479750

[B11] Wheeler QD Meier R (Eds) (2000) Species Concepts and Phylogenetic Theory: A Debate. Colombia University Press, New York.

[B12] Wu ZY (1991) Flora of Yunnan. Science Press, Beijing.

